# The anticancer peptide RT53 induces immunogenic cell death

**DOI:** 10.1371/journal.pone.0201220

**Published:** 2018-08-06

**Authors:** Ewa Pasquereau-Kotula, Justine Habault, Guido Kroemer, Jean-Luc Poyet

**Affiliations:** 1 INSERM UMRS1160, Institut Universitaire d'Hématologie, Hôpital Saint-Louis, Paris, France; 2 Université Paris Diderot, Sorbonne Paris Cité, Paris, France; 3 Equipe 11 labellisée par la Ligue Nationale contre le Cancer, Center de Recherche des Cordeliers, Paris, France; 4 INSERM, Paris, France; 5 Université Paris Descartes/Paris V, Sorbonne Paris Cité, Paris, France; 6 Université Pierre et Marie Curie, Paris, France; 7 Metabolomics and Cell Biology Platforms, GRCC, Villejuif, France; 8 Pôle de Biologie, Hôpital Européen Georges Pompidou, AP-HP, Paris, France; 9 Karolinska Institute, Department of Women's and Children's Health, Karolinska University Hospital, Stockholm, Sweden; 10 c-Dithem, Inserm Consortium for Discovery and Innovation in Therapy and Medicine, Paris, France; China Medical University, TAIWAN

## Abstract

In recent years, immunogenic cell death (ICD) has emerged as a revolutionary concept in the development of novel anticancer therapies. This particular form of cell death is able, through the spatiotemporally defined emission of danger signals by the dying cell, to induce an effective antitumor immune response, allowing the immune system to recognize and eradicate malignant cells. To date, only a restricted number of chemotherapeutics can trigger ICD of cancer cells. We previously reported that a peptide, called RT53, spanning the heptad leucine repeat region of the survival protein AAC-11 fused to a penetrating sequence, selectively induces cancer cell death *in vitro* and *in vivo*. Interestingly, B16F10 melanoma cells treated by RT53 were able to mediate anticancer effects in a tumor vaccination model. Stimulated by this observation, we investigated whether RT53 might mediate ICD of cancer cells. Here, we report that RT53 treatment induces all the hallmarks of immunogenic cell death, as defined by the plasma membrane exposure of calreticulin, release of ATP and the exodus of high-mobility group box 1 protein (HMGB1) from dying cancer cells, through a non-regulated, membranolytic mode of action. In a prophylactic mouse model, vaccination with RT53-treated fibrosarcomas prevented tumor growth at the challenge site. Finally, local intratumoral injection of RT53 into established cancers led to tumor regression together with T-cell infiltration and the mounting of an inflammatory response in the treated animals. Collectively, our results strongly suggest that RT53 can induce *bona fide* ICD of cancer cells and illustrate its potential use as a novel antitumor and immunotherapeutic strategy.

## Introduction

Most anticancer drugs have low therapeutic indices due to their toxicity to normal tissues. Moreover, drug resistance is a recurring problem, emphasizing the need for alternative strategies that selectively and efficiently kill the malignant cell population without affecting normal cells. Recent years have seen much interest in cancer therapies that do not only kill cancer cells but also stimulate, through the emission of danger signals from dying cells, anticancer immunosurveillance, hence inducing a systemic immune response in the host that can control, and even sometimes eliminate neoplastic cells [1–3]. This cell death routine, termed "immunogenic cell death" (ICD), is characterized by the release of damage-associated molecular patterns (DAMPs) and cytokines by the dying cells that mediate chemotactic and adjuvant-like effects, hence eliciting an immune response against tumor-associated antigens [4]. Such DAMPs are sequestered within various subcellular compartments under homeostatic conditions, yet are surface-exposed or released in the context of ICD. Thus, ICD is linked to the exposure of calreticulin and other endoplasmic reticulum proteins at the cell surface [5], as well as the release of ATP [6, 7] and of the non-histone chromatin-binding protein high-mobility group box 1 (HMGB1) [8, 9] into the extracellular milieu. Whereas ICD was originally described as an apoptotic, caspase-dependent form of cellular demise [1, 5], recent data have demonstrated that other forms of cell death, namely necroptosis and necrosis, can also be strongly immunogenic *in vivo*, resulting in long-lasting anti-cancer immunity [10]. Hence, necroptosis induced by chemical dimerization of receptor-interacting protein kinase-3 (RIPK3) or the death domain of Fas-associated death domain (FADD) results in ICD, with necroptotic cancer cells giving a immunogenic preparation in prophylactic tumor vaccination models [11, 12]. In the same line, the oncolytic Newcastle disease virus can induce necroptotic ICD of cancer cells, thus igniting a long-term, tumor-specific immunological memory in an orthotopic mouse model [13]. While freeze–thaw induced necrosis of cancer cells is known to be non-immunogenic [1, 12, 14], it has recently be documented that LTX-315, a synthetic, cationic peptide that permeabilizes the inner mitochondrial membrane, induces a necrotic cell death phenotype exhibiting the hallmarks of ICD [15–18]. Furthermore, when injected into tumors, LTX-315 induced a systemic protective immune response in immunocompetent mice [19–21]. Altogether, these data indicate that several pathways leading to necrosis can lead to ICD.

We previously demonstrated that a peptide comprising the heptad leucine repeat region of the survival protein AAC-11, which acts as a protein-protein interaction domain, fused to the cell-penetrating sequence penetratin selectively killed cancer cells *in vitro* and *in vivo*, while sparing normal cells [22–24]. This peptide, called RT53, can insert into the membranes of cancer cells through interaction with a yet-unknown AAC-11 protein partner. This binding probably allows RT53 to accumulate in cancer cell membranes where it forms pores due to its α-helix, membrane active structure [22]. Interestingly, RT53-treated B16F10 melanoma cells exhibited antitumor vaccination potential in a prophylactic model when mice were subsequently rechallenged with live B16F10 cells [22]. Here, we report that RT53 can induce all the hallmarks of ICD *in vitro* through a non-regulated, lytic mode of action. Interestingly, direct injection of RT53 into established MCA205 fibrosarcomas led to the complete regression of the tumors together with T-cell infiltration and an inflammatory response in an immunocompetent mouse model. These findings reveal the potential of RT53 as a novel antitumor and immunotherapeutic agent.

## Material and methods

### Peptides

All peptides were synthesized by Proteogenix (Strasbourg, France) and were > 95% pure as verified by HPLC and mass spectrographic analysis. Peptides sequences are the following:

RT53: RQIKIWFQNRRMKWKKAKLNAEKLKDFKIRLQYFARGLQVYIRQLRLALQGKT
RT53M: RQIKIWFQNRRMKWKKAKLNAEKLKDFKIRLQYFARGGQVYIRQGRLALQGKT

The penetratin sequence is underlined.

### Cell lines, chemicals and cytochemistry analysis

HUT78 and Lu1205 cells were provided by Drs A. Marie-Cardine and N. Dumaz and genotyped to verify their authenticity. MRC-5 and U2OS cells were provided by Drs M. Dutreix and R. Fåhraeus, and were purchased from ATCC. HaCat cells were provided by Prof. N. Basset-Seguin and their characteristics were described elsewhere [25]. Jurkat and HL-60 were purchased from The European Collection of Cell Cultures. Cells were cultivated either in DMEM or RPMI 1640 (Life Technologies), supplemented with 10% FBS and 1% penicillin/streptomycin. Cytochemistry analysis were performed as previously described [26, 27]. All chemicals were purchased from Sigma.

### Lactate dehydrogenase release, ATP release and HMGB1 release

Release of lactate dehydrogenase (LDH) and ATP in the culture medium were assessed with the CytoTox 96 Non-Radioactive Cytotoxicity Assay and Enliten ATP Assay, respectively (Promega, Madison, WI, USA). HMGB1 release in the culture medium was assessed with the HMGB1 ELISA kit (IBL International, Hamburg, Germany).

### Hemolysis assay

Mice blood was centrifuged at 2000 rpm for 10 min. Red blood cell pellets were washed five times with phosphate-buffered saline (PBS) and resuspended in normal saline. For each assay, 1 × 10^7^ red blood cells were incubated with or without peptide (30 μM) at 37°C for 1h. The samples were centrifuged at 4500 rpm for 5 min and the absorbance of the supernatant was measured at 540 nm. To determine the percentage of lysis, absorbance readings were normalized to lysis with 1% Triton X-100.

### Electronic microscopy

Samples were fixed in 3% glutaraldehyde in phosphate buffer, pH 7.4 for 1 hour, washed, post-fixed with 1% osmium tetroxide in 0.1 M phosphate buffer and then gradually dehydrated in 70, 90 and 100% ethanol. After 10 min in a 1:2 mixture of epoxy propane and epoxy resin and 10 min in epon, samples were embedded in epoxy resin and polymerized at 60°C for 24 h. After polymerisation, ultrathin sections of 90 nm were cut with an ultra-microtome (Reichert ultracut S), stained with uranyl acetate and Reynold’s lead and observed with a transmission electron microscope (JEOL 1011). Acquisition was performed with a Gatan Orius 1000 CCD camera.

### Visualization of calreticulin-GFP aggregation

U2OS cells stably expressing a calreticulin-GFP fusion protein were described elsewhere [28]. Cells grown on coverslips were washed twice with PBS, fixed in PBS containing 4% paraformaldehyde and imaged using a Zeiss Axiovert 200 M microscope.

### Western-blotting

Cells were lysed in 50 mM Tris/HCl pH 7.6, 150 mM NaCl containing 1% Triton-X100, 10 μg/ml leupeptin, 10 μg/ml aprotinin and 0.1 mM phenylmethylsulfonyl fluoride (PMSF) and clarified by centrifugation at 15,000 g for 15 min. Proteins were resolved by SDS PAGE and transferred onto a polyvinylidene difluoride (PVDF) membrane (Bio-Rad). For Western blot analysis, membranes were blocked in PBS, 0.1% Tween 20, 5% BSA for 1 h followed by incubation overnight at 4°C with one of the following rabbit polyclonal antibodies: anti-eIF2α (cat #9722) or anti-phospho-eIF2α (Ser51, cat #9721) (Cell Signaling). After being washed three times in PBS, 0.1% Tween 20, membranes were incubated for 1 h with goat anti-rabbit horseradish peroxidase-conjugated IgG (Sigma) in blocking solution. Membranes were then washed extensively in PBS, 0.1% Tween 20 and developed by using enhanced chemiluminescence detection reagents (Amersham Biosciences) in accordance with the manufacturer’s protocol.

### Ethics statement

This study has been carried out in accordance with the EC Directive 86/609/EEC for animal experiments and was approved by the Committee for Experimental Animal Studies of the University of Paris 7 Institute Board Ethics (Protocol Number: 2303.01). Animals were housed in vented animal cabinets under controlled temperature (22°C) and 12 h light-dark cycle under pathogen-free conditions and were allowed food and water *ad libitum*. Prior to the start of the experiments, mice were allowed to rest for 1 week following shipment. All efforts were made to minimize suffering. Animals were euthanized by cervical dislocation under anesthesia with 3% isoflurane. Female C57BL/6 mice were from ENVIGO (France).

### Tumor vaccination assay

MCA205 cells were harvested using Versene (Invitrogen), washed in PBS and resuspended in 200 μl of serum-free RPMI medium. The cells were then exposed to 30 μM RT53 for 3h for cell death induction and the whole suspension of RT53-treated cells was injected subcutaneously (2x10^6^ cells) into the left flanks of C57BL/6 mice (*n* = 6 per group). Eight days later, the mice were challenged subcutaneously on the right flank with 0.5x10^6^ live MCA205 cells. Tumor growth on the challenge site was evaluated using a digital caliper and volume was calculated using the formula: Length x Width^2^/2. Animals were euthanized by cervical dislocation under anesthesia with 3% isoflurane when tumor size reached the ethical end point or were necrotic.

### Intratumoral treatment

Mouse xenograft tumors were obtained by subcutaneous injection of 0.5x10^6^ MCA205 cells into the right flanks of C57BL/6 mice (*n* = 6 per group). When tumors reached a size of 20–40 mm^3^, the mice received intratumoral injection of 300 μg of RT53 or vehicle (normal saline) for three consecutive days. Tumor growth was evaluated using a digital caliper and volume was calculated using the formula: Length x Width^2^/2. Animals were euthanized by cervical dislocation under anesthesia with 3% isoflurane when tumor size reached the ethical end point or were necrotic. Following anesthesia, xenografts were removed for immunohistochemical staining and cytotoxicity analysis.

### Histological analysis

Histological Tumors were fixed in 4% neutral buffered formalin and embedded in paraffin. Sections (4μm) were stained with hematoxylin-eosin (H&E) and subjected to microscopic analysis. To investigate T cells infiltration, sections were stained with an anti-CD3 antibody (Dako, ref: A0452) or rabbit IgG isotype control. Histological analysis was performed at the HistIM facility of Cochin Institute (Paris, France). Slides were imaged using a Lamina multilabel slide scanner (Perkin Elmer). For quantitative analysis of T cells infiltration, six different and noncontiguous representative fields (40x magnification) were randomly selected for each experiment and their areas were quantified for immunoreactive CD3.

### RNA extraction and real-time PCR

RNA was extracted from tumors using the Qiagen Rneasy Mini kit, according to the manufacturer’s instructions, and was reverse transcribed using the High Capacity cDNA Reverse Transcription Kit (Applied Biosystems) using random hexamers. Quantitative PCR was performed with the StepOne real-time PCR system (Applied Biosystems) using Perfecta SYBR Green FastMix, ROX (Quanta Biosciences, Beverly, MA, USA) and the following primers: CCL2, F 5’-CTCAGCCAGATGCAGTTAACG-3’, R 5’-GCTGCTGGTGATCCTCTTGT-3’; CXCL10, F 5’- CCTGCCCACGTGTTGAGAT-3’, R 5’-TGATGGTCTTAGATTCCGGATTC-3’; GAPDH, F 5’-CATGTTCCAGTATGACTCCACTC-3’, R 5’- GGCCTCACCCCATTTGATGT-3’. Melting curve analyses were performed to verify the amplification specificity. Relative quantification of gene expression was performed according to the comparative CT (ΔΔCT) method using StepOne Software 2.0 (Applied Biosystems).

### ELISA analysis of plasma

Blood from C5BL/6 mice with tumor size of 20–40 mm^3^ was harvested at the selected time points following single intratumoral injection of 300 μg of RT53 or vehicle (normal saline). *In vivo* secretion of IL-1β and IL-6 in the serum was assessed using the Mouse IL-1β/IL-1F2 Quantikine ELISA Kit and Mouse IL-6 Quantikine ELISA Kit, respectively, per the manufacturer's instruction (R&D System).

### Statistical analysis

Student's *t* test was used to test for statistical significance of the differences between the different group parameters. *p* values of less than 0.05 was considered statistically significant.

## Results

### RT53 induces rapid cancer cells necrosis

**Fig 1:**
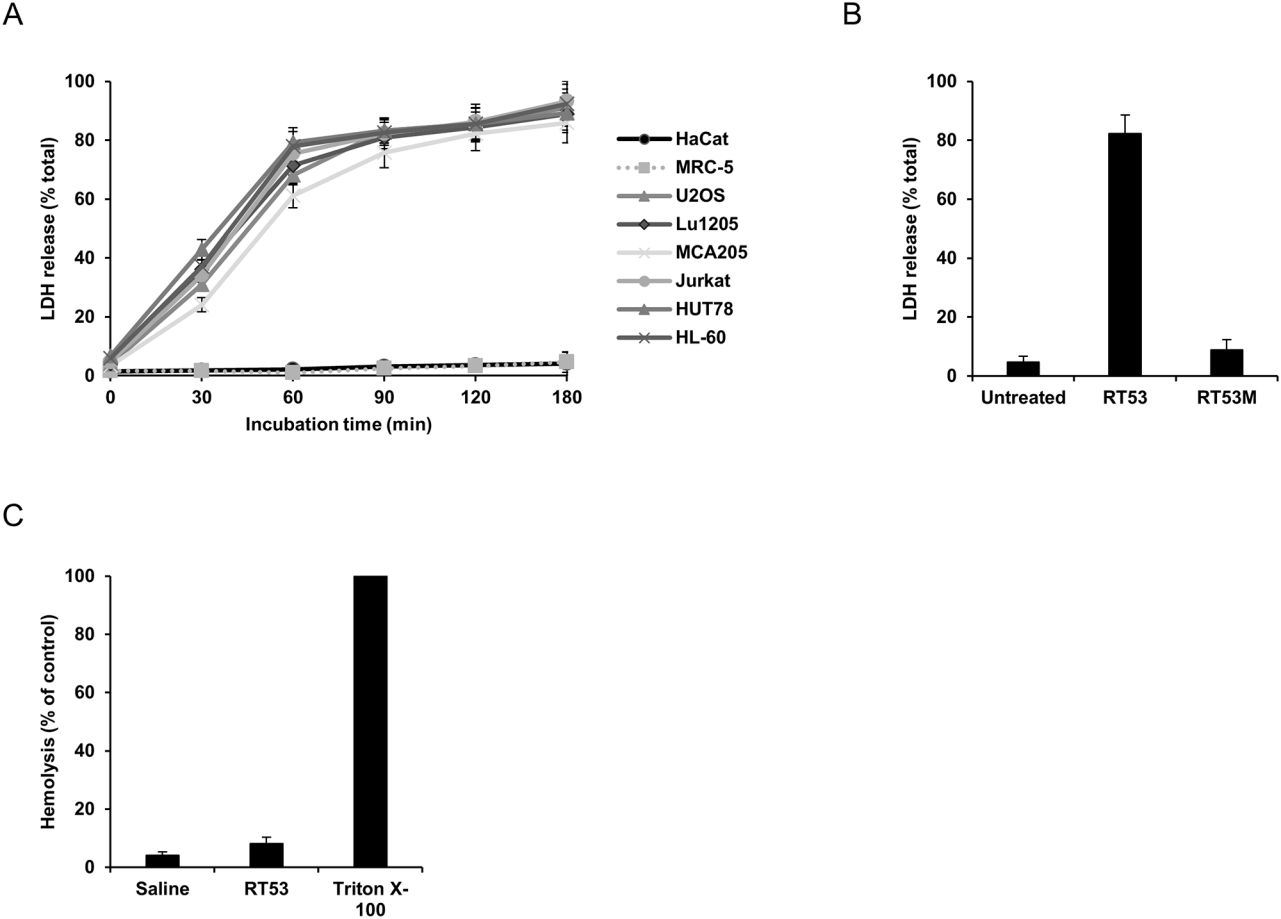
RT53 induces rapid cancer cells membranolysis. (A) Non-malignant (HaCat and MRC-5) and cancerous (U2OS, Lu1205, MCA205, Jurkat, HUT78 and HL-60) cells were treated with 20 μM of RT53. Plasma membrane permeability was evaluated at the designed time points by measuring extracellular LDH into the culture medium. The obtained values were normalized to those of the maximum LDH released (completely lysed) control. Data are means±s.e.m. (*n* = 3) (B) U2OS cells were treated with 20 μM of RT53 or the control peptide RT53M for 3 h. Plasma membrane permeability was evaluated as in (A). (C) Mice red blood cells were incubated with 20 μM of RT53. Released hemoglobin was detected by densitometry at 540 nm. Hemoglobin release by cells treated with 1% Triton X-100 was used as 100% lysis control.

**Fig 2:**
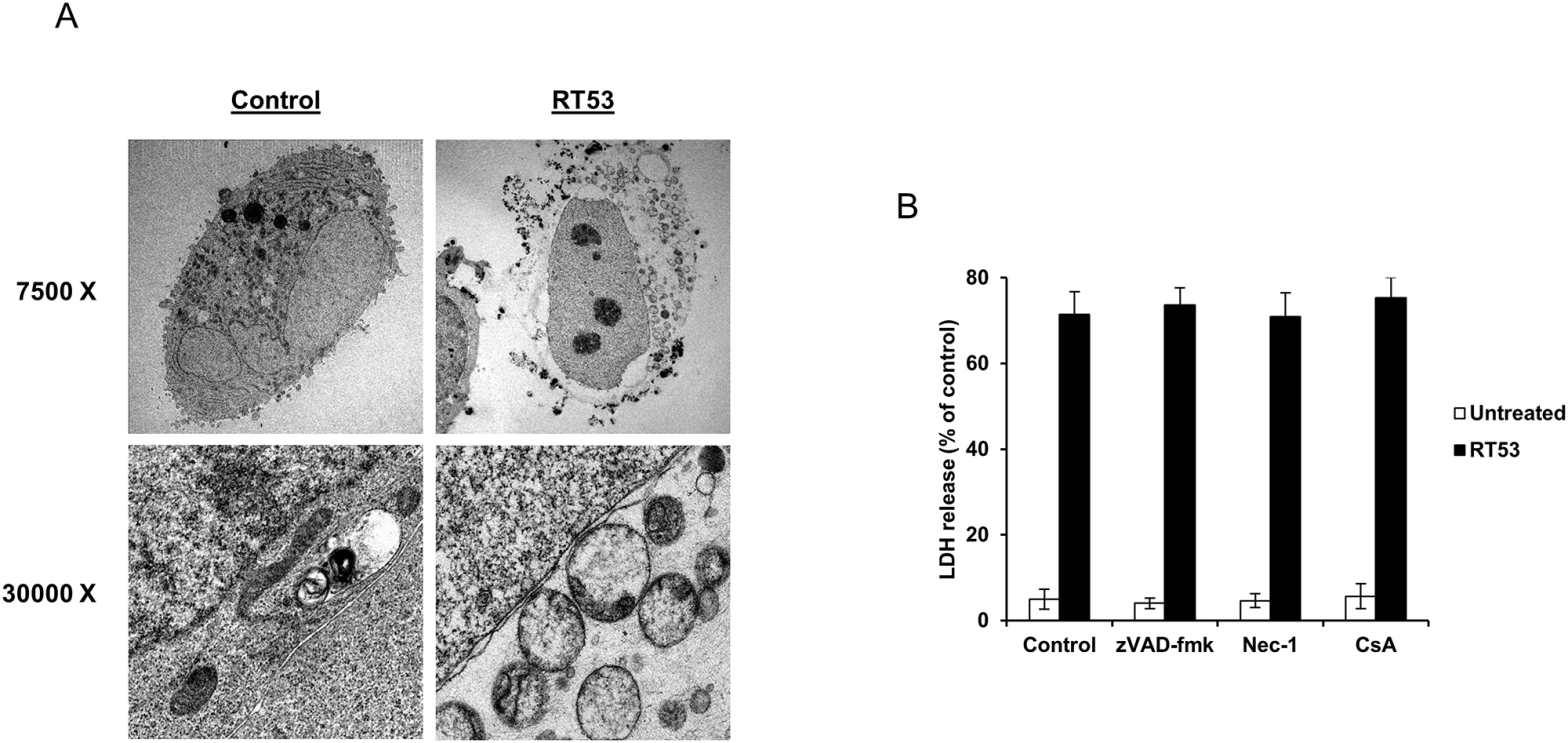
RT53 induces unregulated necrotic cell death. (A) Ultrastructural analysis of RT53-mediated cell death. U2OS cells were left untreated (Control) or exposed to 15 μM of RT53 for 30 min. Cells were then analyzed by transmission electron microscopy following osmium tetroxide staining. (B) U2OS cells were exposed to 20 μM of RT53 in the presence or absence of 50 μM zVAD-fmk, 50 μM Necrostatin-1 (Nec-1) or 100 mM cyclosporin A (CsA) for 1 h. Necrotic cell death was monitored by lactate dehydrogenase (LDH) release from cells into the culture medium. The obtained values were normalized to those of the maximum LDH released (completely lysed) control. Data are means±s.e.m. (*n* = 3).

We first investigated which cell death modality is induced by RT53. Kinetic experiments indicated that, when added to various human or mouse cancer cell lines, RT53 induced the rapid loss of plasma membrane integrity, as detected by the release of the intracellular enzyme lactate dehydrogenase (LDH) (Fig 1A). However, no cytotoxic effects were observed when RT53 was tested on the non-malignant cells HaCat and MRC-5 (Fig 1A). In line with our previous observations [22], a mutant peptide (RT53M) in which leucines 384 and 391 (AAC-11 numbering) were substituted by alanines did not affect the integrity of the plasma membrane (Fig 1B). Importantly, a hemolytic assay revealed that RT53 treatment did not cause erythrocyte lysis (Fig 1C), ruling out an unspecific detergent-like effect for RT53 as sometimes observed for amphiphilic peptides [29]. Altogether, these data indicate that the RT53-induced plasma membrane permeabilization is specific to cancer cells and appears to occur *via* a defined structural target(s). We next further evaluated the mode of action underlying RT53 cytotoxic activity by means of transmission electron microscopy analysis. Observation of U2OS osteosarcoma cells treated with RT53 revealed an obvious necrotic morphology, with a disrupted plasma membrane and subsequent release of intracellular materials (Fig 2A). Interestingly, nuclei from RT53-treated cells appeared largely intact, without evidence of chromatin condensation, indicating the RT53-mediated cell death does not involve a direct form of conventional apoptosis but rather a membranolytic mode of action. In line with this hypothesis, the pan-caspase inhibitor Z-VAD-fmk failed to prevent membrane permeabilization by RT53, as assessed by the release of lactate dehydrogenase (LDH) into the culture medium (Fig 2B). Regulated necrosis includes multiple cell death routes such as necroptosis or mitochondrial permeability transition pore (MPTP)-mediated necrosis. However, inhibition of necroptosis by necrostatin-1 or blockade of the MPTP by cyclosporin A failed to interfere with RT53-mediated loss of membrane integrity (Fig 2B). Therefore, these results indicate that the cytotoxic effects of RT53 toward cancer cells occur mainly through a non-regulated form of necrosis.

### RT53 elicits ICD *in vitro*

**Fig 3:**
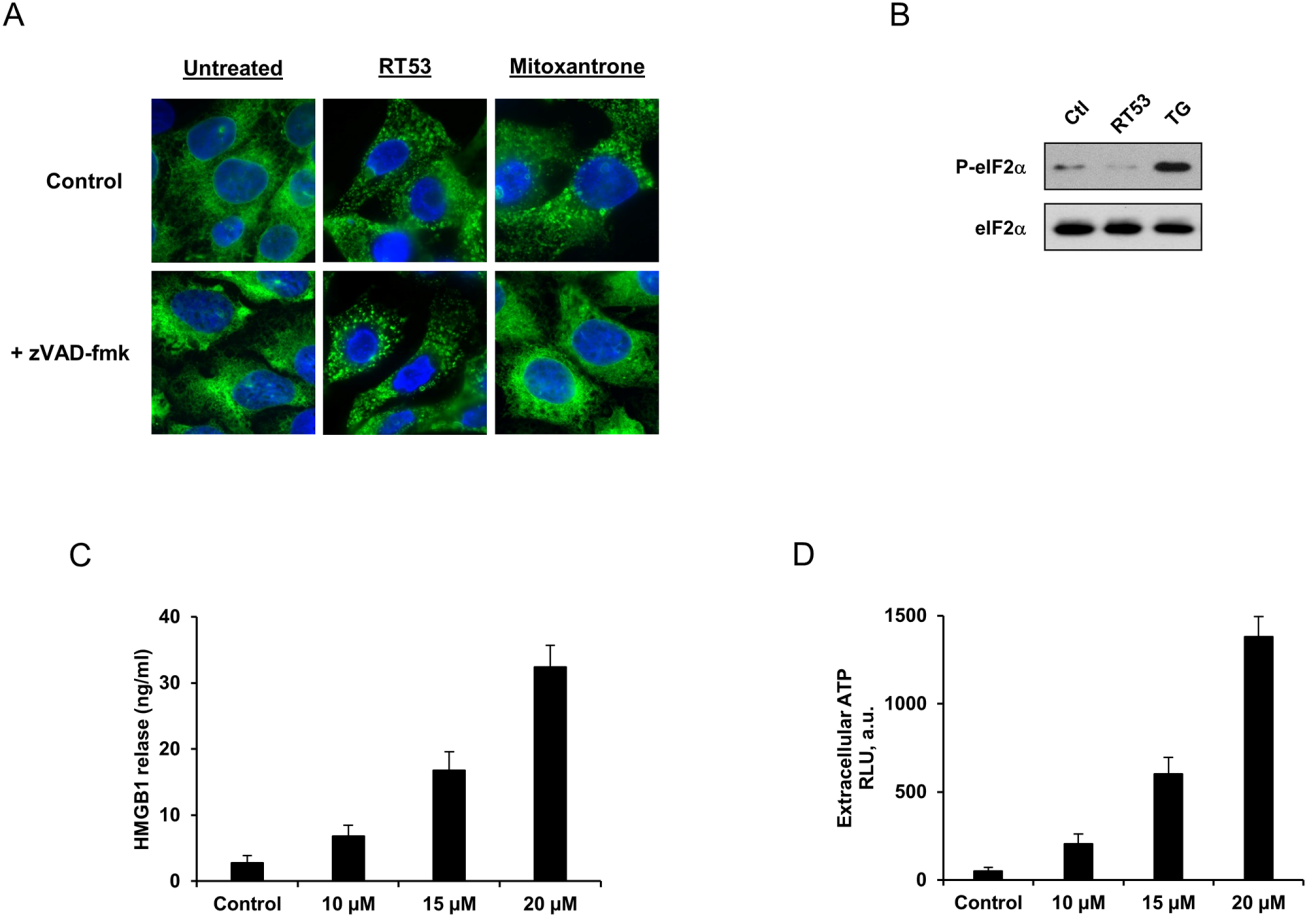
RT53 triggers calreticulin exposure as well as the release of HMGB1 and ATP. (A) U2OS cells stably expressing CRT-GFP were treated with 10 μM of RT53 or 1 μM mitoxantrone for 6 h, in the presence or absence of zVAD-fmk. Cells were then, fixed, stained for DNA, and examined by fluorescence microscopy. (B) U2OS cells were left untreated or treated with 10 μM of RT53 or 200 nM thapsigargin (TG) as a positive control for 6 h. Cell lysates were analyzed by Western blot for phosphorylated and total protein eIF2α. (C) U2OS cells were left untreated or exposed to increasing concentrations of RT53 for 3h. Extracellular HMGB1 was then measured in the culture supernatant. Data are means±s.e.m. (*n* = 3). (D) U2OS cells were left untreated or exposed to increasing concentrations of RT53 for 3h. Extracellular ATP was then measured in the culture supernatant. Data are means±s.e.m. (*n* = 3).

As recent evidence suggests that necrosis may be immunogenic [15–21], we investigated whether RT53 treatment would be able to induce the hallmarks of ICD, which include the surface exposure of the endoplasmic reticulum (ER) chaperone calreticulin (CRT) and the release of ATP and HMGB1 [30]. Surface exposure of CRT by tumor cells constitutes a major checkpoint of ICD, because CRT favors their engulfment by dendritic cells [5, 31]. CRT aggregation has been reported to precede its plasma membrane exposure [32]. Using a defined biosensor model [28, 33] of U2OS cell line stably expressing a CRT-green fluorescent protein (GFP), we observed the redistribution of the CRT-GFP fusion protein from a near-to-diffuse to a granular pattern in response to RT53 (Fig 3A). Of note, whereas CRT exposure induced by chemotherapeutic agents such as mitoxantrone requires caspase-8 activation [34], the pan-caspase inhibitor zVAD-fmk failed to affect calreticulin aggregation induced by RT53. As an internal control, zVAD-fmk efficiently prevented CRT-GFP aggregation induced by the ICD inducer mitoxantrone (Fig 3A). Moreover, whereas chemotherapy-driven CRT translocation relies on phosphorylation of the eukaryotic transcription factor eIF2α [5], RT53 exposure did not induce phosphorylation of eIF2α (Fig 3B). Therefore, our data indicate that in contrast to the “canonical” (chemotherapy-elicited) CRT pathway, RT53 can trigger CRT exposure in a caspase- and eIF2α-independent pathway. We next tested whether RT53 would stimulate the release of the two DAMPs HMGB1 and ATP, two obligatory signals of immunogenicity. As shown in Fig 3C, RT53 treatment of U2OS cells triggered the release of HMGB1 into the culture medium, as detected by ELISA assay, in a dose-dependent manner. Moreover, ATP-bioluminescence assays revealed that RT53 treatment resulted in a dramatic increase of extracellular ATP (Fig 3D), demonstrating that RT53 can promote the secretion of both DAMPs by cancer cells. Taken together, these results indicate that RT53 can induce all surrogate markers of ICD *in vitro* in an apoptosis-independent manner.

### RT53-treated cancer cells or intratumoral RT53 administration have antineoplastic effects *in vivo*

**Fig 4:**
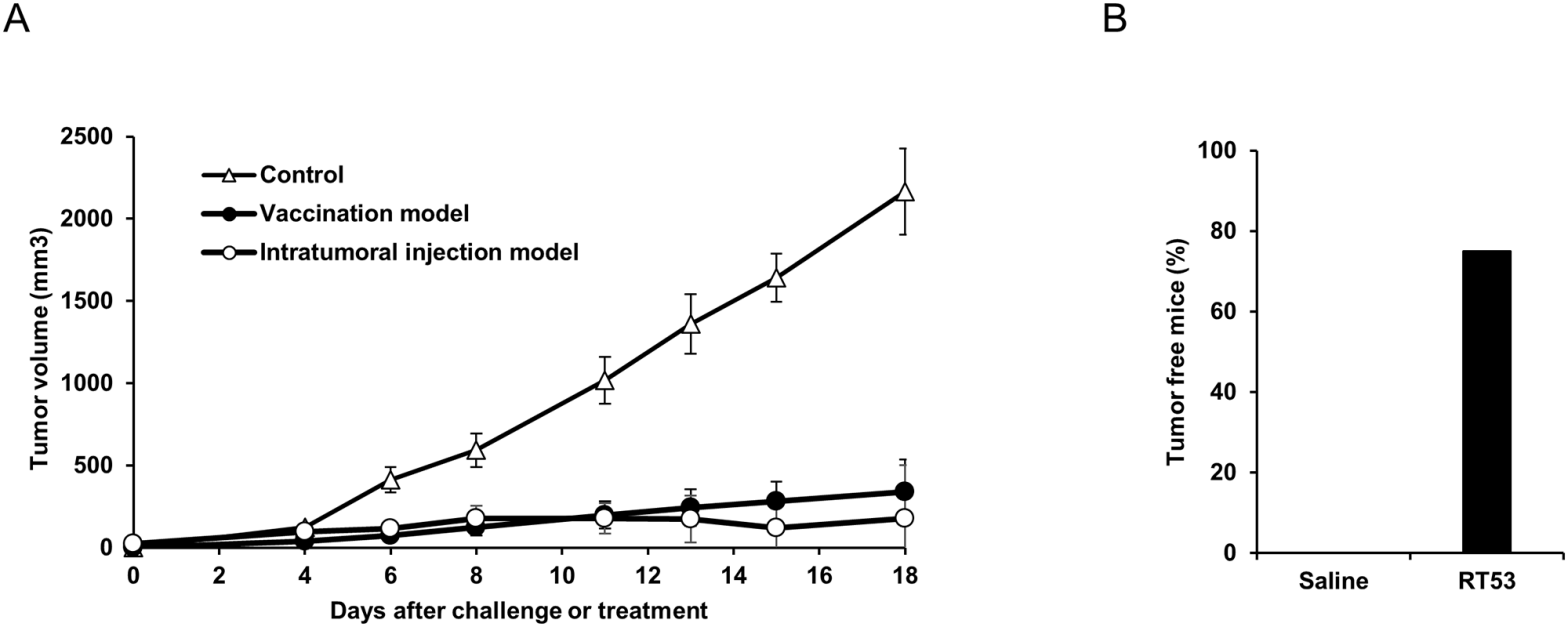
RT53 treatment prevents tumor growth. (A) Closed circle: RT53-treated cancerous cells induce anti-tumor immunogenicity in a prophylactic tumor vaccination assay. Syngeneic C57BL/6 mice were injected s.c. on the left flank with 2x10^6^ MCA205 cells treated *in vitro* with RT53. Eight days later, the mice were challenged subcutaneously on the right flank with 0.5x10^6^ live MCA205 cells and tumor growth was monitored. (*n* = 6 per group). Open circles: effect of intratumoral injection of RT53 on the growth of established tumors. Palpable MCA205 tumor on C57BL/6 mice were injected intratumorally with 300 μg RT53 once a day for 3 days. Open triangles represent the growth in saline-treated controls. Tumor growth was monitored on day 1 of injections (left panel). (*n* = 6 per group). (B) Percentage of tumor-free mice at day 21.

Since RT53 induces all major characteristics of ICD *in vitro*, we next assessed the antitumor effects of the peptide using well-characterized syngeneic tumor models. In a first experiment, we evaluated the ability of RT53-treated cells to induce anti-tumor immunity in a prophylactic tumor vaccination model. As shown in Fig 4A, immunization of C57BL/6 mice by subcutaneous injection of MCA205 fibrosarcoma cells treated *in vitro* by RT53 subsequently resulted in drastically reduced tumor growth at the challenge site compared to control mice. This result thus strongly suggests that RT53 treatment of cancer cells can induce an adaptive immune response *in vivo*. To further evaluate the ability of RT53 to elicit an antitumor response, we investigated the therapeutic effect of direct administration of the peptide to tumors. For that purpose, RT53 was injected once a day for three consecutive days into established MCA205 fibrosarcomas growing in C57BL/6 mice. As shown in Fig 4A, intratumoral administration of RT53 resulted in a nearly complete suppression of tumor growth. Notably, complete tumor regression was observed in 75% of the mice receiving the treatment (Fig 4B). Importantly, no weight loss or obvious clinical symptoms were observed in the treated animals (not shown). Therefore, these data indicate that RT53 can both act locally and as an immunogenic cytotoxic compound *in vivo*.

### Intratumoral administration of RT53 induces massive tumor necrosis, T cell infiltration and an inflammatory response

**Fig 5:**
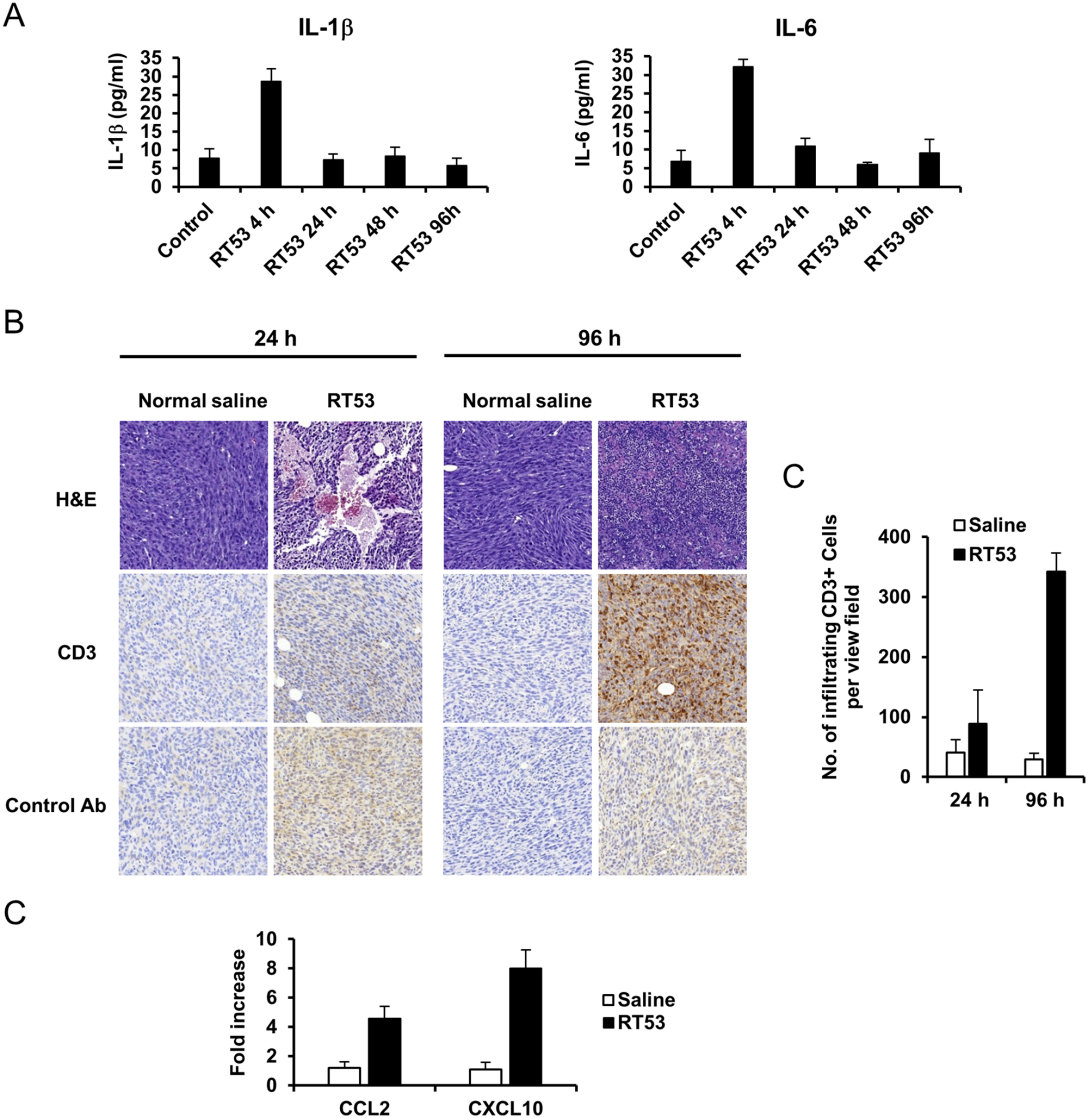
Intratumoral administration of RT53 induces tumor necrosis, immune cells infiltration and inflammation. (A) Plasma was harvested 4, 24, 48 or 96 h following single intratumoral injection of normal saline (control) or 300 μg RT53 in normal saline and IL-1β (left panel) as well as IL-6 (right panel) levels were estimated using ELISA. Data from 3 animals are presented for each time point as mean±SEM. (B) Established MCA205 fibrosarcomas were surgically excised 24 h or 96 h post intratumoral injection with normal saline (control) or 300 μg RT53 in normal saline and sections subjected to H&E staining (top) or stained for CD3 or rabbit IgG isotype control (middle and bottom, respectively). (C) Number of infiltrating CD3-positive cells per view field following CD3 staining. Data from 3 animals are presented as mean±SEM. (D) Relative transcription of CCL2 and CXCL10 (normalized to GAPDH) as determined by real-time RT-PCR on samples of tumors 24 h after RT53 or normal saline injection. Values of CCL2 and CXCL10 are represented as fold change relative to untreated tumors, set to 1 (mean±SEM; n = 3).

To investigate the underlying mechanisms behind the antitumor effects of RT53, we first tested whether intratumoral injection of RT53 could induce an inflammatory response. For that purpose, plasma from RT53-treated or control animals was analyzed for IL-1β and IL-6 levels. As shown in Fig 5A, both IL-1β and IL-6 levels sharply increased 4 h following intratumoral RT53 injection compared to controls. This increase in cytokine levels was not persistent, as no differences in IL-1β and IL-6 levels were noted from 24 h up to 96 h following RT53 administration (Fig 5A). In order to assess histological changes resulting from local injection with RT53, sections of MCA205 tumors treated with a single intratumoral administration of normal saline or RT53 and harvested 24 h or 96 h following treatment were analyzed. Hematoxylin–eosin staining revealed dramatic hemorrhagic tumor necrosis in RT53-treated tumors, whereas tumors injected with normal saline exhibited minimal necrosis. (Fig 5B top and S1 Fig). As observed for the lytic peptide LTX-315 [17], tumor necrosis was clearly noticeable 24 h post injection. Interestingly, immunohistochemical analysis revealed massive infiltration by CD3^+^ T lymphocytes in the RT-53 treated tumors 96 h post injection, while control tumors contained a rather low number of T cells (Fig 5B middle, Fig 5C and S2 Fig). To evaluate changes in the tumor microenvironment induced by RT53 injection that could explain the increased number of T lymphocytes, we used real-time RT-PCR of tumor extracts to analyze the relative expression of the pro-inflammatory chemokine ligands CCL2 and CXCL10. As shown in Fig 5D, the levels of both CCL2 and CXCL10 transcripts were markedly increased upon RT53 intratumoral injection compared to saline-injected tumors. Altogether, these results indicate that, in addition to the local immune response, intratumoral RT53 injection triggers a transient systemic inflammatory response accompanied by the elevation of circulating cytokines.

## Conclusion

A number of mechanisms have been proposed to account for the failure of chemotherapies in cancer patients. Beside the development of drug resistance by malignant cells, tumors can also escape recognition and clearance by the immune system through the activation of multiple immunosuppressive mechanisms [35]. Recent observations have revealed that, among the cytotoxic chemotherapeutic agents used in clinic, some were able to induce an immunogenic form of apoptosis as the succumbing cancer cells could induce a potent antitumor immune response [36]. This so-called immunogenic cell death (ICD) is now known to increase the immunogenicity of dying cancer cells owning to the release of endogenous danger signals or damage-associated molecular patterns (DAMPs) [37] that alert the innate immune system, resulting in an effective antitumor immune response. As the immune system plays a fundamental role in long-term tumor control, great efforts are currently undertaken to elaborate chemotherapeutic approaches that can elicit immunogenic cancer cell death. We have previously reported that the penetrating peptide RT53, based on the fusion of the heptad leucine repeat region of the survival protein AAC-11 (residues 363–399) and the penetratin sequence, induces cancer cell death *in vitro* and inhibits melanoma tumor growth in a xenograft mouse model [22]. In the present study, we demonstrate that RT53 selectively induces necrosis of cancer cells and that this necrotic cell death is immunogenic. RT53-induced cancer cells death occurred through an apoptosis-independent, membranolytic mechanism, as evidenced by the release of the cytoplasmic enzyme lactate dehydrogenase (LDH) as well as transmission electron microscopy. Here, a necrotic phenotype with no morphological signs of apoptosis, including nuclear condensation, was clearly evident. Accordingly, blockage of apoptosis by means of caspases inhibition failed to prevent the RT53-induced killing of cancer cells. Further experiments revealed that RT53 did not induce a typical regulated necrosis, as neither necrosatin-1, which inhibits receptor-interacting protein kinase (RIPK) 1-mediated necroptosis, nor cyclosporine A, which inhibits mitochondrial permeability transition-mediated regulated necrosis, interfered with cell death induction by RT53. Therefore, our results indicate that RT53-induced necrosis occurs in a passive manner, upon plasma membrane destruction. Our previous results indicate that RT53 accumulates within the plasma membrane of cancer cells, but not to that of non-cancerous cells [22]. Moreover, we show here that RT53 failed to lyse erythrocytes or normal cells, indicating that RT53-mediated cancer cells necrosis does not merely result from an unspecific, detergent-like membrane permeabilization. Although we cannot exclude a role for the physicochemical characteristics of cancer cells membranes, we hypothesize that RT53 targets a yet unknown cancer cell-specific membrane-bound partner(s), as observed for several described cancer cell-specific, membranolytic peptides [38–40]. This binding likely increases the local concentration of RT53, resulting in membrane lysis, owing to its membrane active, alpha helix, structure [22]. This selective and rapid tumor membrane disruptive behavior is consistent with other investigations that have examined the mechanism of action of other pore forming anticancer peptides [41]. Interestingly, our study indicates that RT53-induced cell death is accompanied by all the hallmarks of ICD *in vitro*, namely the translocation of calreticulin to the cell surface and the extracellular release of HMGB1 and ATP. These three DAMPs act as potent immunostimulants and are known to possess a key role in the immunogenicity of nearly all ICD inducers [2]. Whereas HMGB1, which is a chemotactic for immune cells and elicits adaptive immunity, and ATP, which acts as a “find me” signal, can be passively released from damaged cells [42, 43], calreticulin exposure on dying cells usually is the result of a complex signaling process [44]. Surface-exposed calreticulin is a crucial DAMP in ICD, as it dictates tumor specific immunity, allowing the recognition and engulfment of tumor cells by dendritic cells [5, 31]. Noteworthy, unlike the “canonical” (chemotherapy-induced) calreticulin exposure pathway, RT53-induced calreticulin exposure was found to be both caspase- and eIF2α-independent, similar to what observed with photodynamic therapy and high hydrostatic pressure induction of ICD [45, 46]. Therefore, our results indicate that the passive cell death caused by RT53 is fully able to allow the activation of the key DAMPs *in vitro*.

Importantly, RT53 treatment appears to induce *bona fide* ICD as RT53-exposed cells could serve as potent immunizers in a vaccination assay. Indeed, inoculation of RT53-killed fibrosarcomas cells into syngenic, immunocompetent mice resulted in a drastic reduction of tumor growth at the challenge site, when using MCA205 living cells. These results are in line with those obtained using another experimental animal model [22] and suggest that RT53 does mediate anti-tumor immunity *in vivo*. To further assess the anticancer properties of RT53, we evaluated the effects of intratumoral administration of the peptide. Interestingly, the majority of treated animals displayed a complete suppression of tumor growth. Based on our findings that RT53 induces tumor cell necrosis *in vitro*, we hypothesized that the tumor growth inhibition we observed *in vivo* upon local RT53 injection also occurred by this mechanism. Indeed, histological examinations revealed that administration of RT53 caused massive hemorrhagic necrosis, indicating that direct injection of RT53 into tumors results in total cell killing and tumor growth blockade and regression. Moreover, local injection of RT53 was followed by an increased infiltration of T cells, suggesting that the direct oncolytic effect of RT53 leads to the generation of a more immunogenic environment that promotes the infiltration of lymphocytes. Immune cells infiltration into tumors is governed by chemotaxis, with the local chemokine network influencing the numbers and types of lymphocytes recruited [47]. In particular, the chemokines CCL2, CCL3, CCL4, CXCL9 and CXCL10 are known to be potent chemoattractants for tumor-infiltrating T cells [48] and increased expression of these chemokines has been shown to be associated with infiltration of tumor-specific T cells in cancer patients and various mice models of cancer [47–54]. Interestingly, our data demonstrate an upregulation of the genes encoding CCL2 and CXCL10 in tumors following RT53 injection. Therefore, our data suggest that enhanced production of these chemokines may explain the observed increased infiltration of T cells into the RT53-treated tumors. Several cell types within the tumor microenvironment can be involved in the secretion of specific chemokines, including the tumor cells themselves, macrophages, endothelial cells or the recruited T cells [48, 55]. It will be of interest to characterize the cell types producing the detected chemokines, as well as the tumor-infiltrating T cell populations upon RT53 injection. Intratumoral administration of RT53 led to a marked increase of the cytokines IL-1β and IL-6 levels in the circulation 4 h post injection. The upregulation of these cytokines indicates that local injection of RT53 stimulates pro-inflammatory processes and the systemic activation of the immune system.

Our results are reminiscent to those obtained when studying the anti-cancer effects of another membranolytic peptide, LTX-315. Like RT53, LTX-315 induces an unregulated form of necrosis that can lead to the release of DAMPs *in vitro* and the induction of a potent immune response in different preclinical tumor models [16–19, 21, 56, 57]. As accidental necrosis of cancer cells provoked by freeze-thawing or boiling fails to induce a potent immune response *in vivo* [1, 11], our results, combined to those obtained with LTX-315, indicate that the induction of ICD obtained with these oncolytic peptides is not a consequence of the mere spillage of intracellular components into the extracellular milieu, and that ICD is not restricted to immunogenic apoptosis. However, more work is still needed to characterize the precise molecular mechanisms that underlie RT53-induced ICD. In particular, it will be interesting to explore the mechanisms through which intratumoral administration of RT53 shifts the tumor microenvironment towards local immunostimulation.

In recent years, the concept that curative cancer therapy requires the activation of an antitumor immune response by the dying cancer cells has emerged [2]. To date, only few chemotherapeutics can trigger ICD. Therefore, our results indicate that RT53 might constitute a promising immunotherapeutic agent.

## Supporting information

S1 Fig Intratumoral administration of RT53 induces tumor necrosis. Established MCA205 fibrosarcomas where surgically excised 24 h or 96 h post intratumoral injection with normal saline (control) or 300 μg RT53 in normal saline and sections subjected to H&E staining.
(TIF)

S2 Fig Intratumoral administration of RT53 induces immune cells infiltration. Established MCA205 fibrosarcomas where surgically excised 24 h or 96 h post intratumoral injection with normal saline or 300 μg RT53 in normal saline and sections subjected to CD3 staining. For quantitative analysis of T cells infiltration, 6 different and noncontiguous representative fields (40x magnification) were randomly selected for each experiment and their areas quantified for immunoreactive CD3.
(TIF)

S1 File NC3Rs ARRIVE guidelines checklist. (PDF)
