# Supplementary figures and images for: The anticancer peptide RT53 induces immunogenic cell death
Source: PLoS One. 2018 Aug 6;13(8):e0201220. doi: 10.1371/journal.pone.0201220 (PMC6078289; doi:10.1371/journal.pone.0201220)

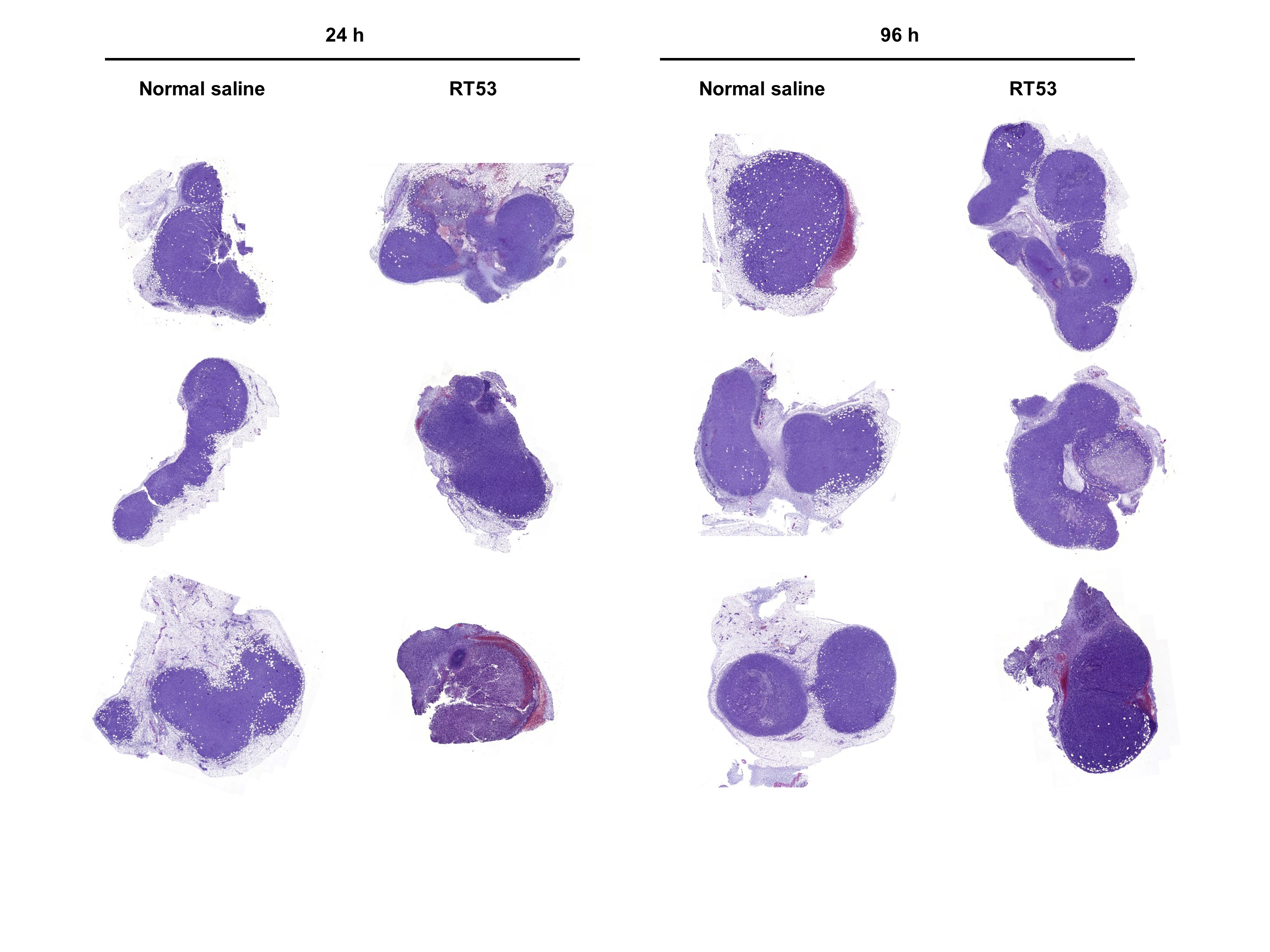

Supplement: S1 Fig — Established MCA205 fibrosarcomas where surgically excised 24 h or 96 h post intratumoral injection with normal saline (control) or 300 μg RT53 in normal saline and sections subjected to H&E staining. (TIF) [file pone.0201220.s001.tif]

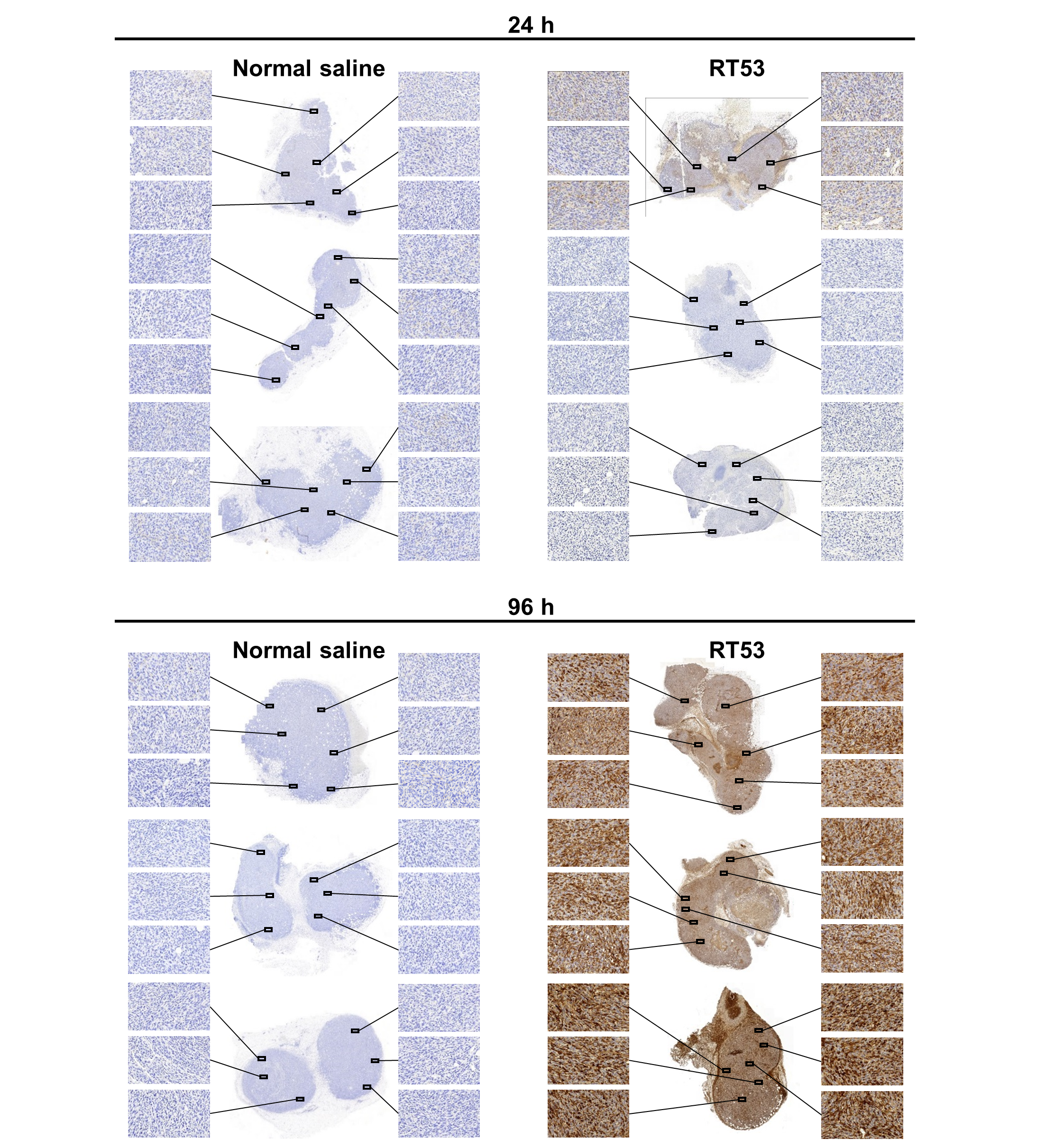

Supplement: S2 Fig — Established MCA205 fibrosarcomas where surgically excised 24 h or 96 h post intratumoral injection with normal saline or 300 μg RT53 in normal saline and sections subjected to CD3 staining. For quantitative analysis of T cells infiltration, 6 different and noncontiguous representative fields (40x magnification) were randomly selected for each experiment and their areas quantified for immunoreactive CD3. (TIF) [file pone.0201220.s002.tif]
